# Miscellanies

**Published:** 1833-10-01

**Authors:** 


### Miscellanies.

### I. Aldersgate Dispensary.

We little expected that, in the nineteenth century, and after the general feelings of the English nation had given birth to a reformed Parliament, we should see a return of the worst and most venal days of ancient Rome! The Prætorian bands put up the imperial throne to public auction, and it was knocked down to the highest bidder! So, the majority of governors of the Aldersgate Dispensary, in imitation of the Prætorian bands, have passed a law, by which the fullest purse, with the emptiest head, may fill the most important medical and surgical offices of the institution! If the governors had fairly and unequivocally stated that each new appointment would be publicly sold, for the *benefit* of the institution, they would have been entitled to the credit of candour, to palliate an act of suicidal mania—to give it the most gentle term; but when they enact a sham law against bribery and corruption in the elections, knowing, as they do, that it is only a mockery and an insult to common sense, they are entitled to no other appellation than that which we have given them! In the simplicity of their hearts, they thought that a rich harvest would be reaped, at every vacancy, by the bribery system; whereas, the open acknowledgement of such a system will destroy all competition, and there will not be a single bribe offered at each election. The honour of the medical profession is now put to a trial. We hope that a few thoughtless members will not compromise its dignity. If they do, they run a fearful risk—they take upon themselves a most dangerous responsibility. No time or circumstance can ever wipe away the stigma that will attach to physicians or surgeons, who step in to take office under such a system of open bribery and corruption. It is no palliation or excuse, that such a law should have existed at the beginning of an institution, when funds were to be raised to give it impulse. Such a law was never defensible—and, after being revoked, it is insulting to the moral sense of mankind to renew it. Those medical officers who become candidates for the situations resigned, should recollect that they are betraying the character of their brethren at large; for what will the world think of that profession, which rushes voluntarily to the support of a law the most iniquitous, profligate, and venal, that could well have been devised? They will injure themselves—injure the institution—and, what is worse, they will injure the whole faculty in the eyes of every honest man! Too much praise cannot be given to the late medical officers of the Aldersgate Dispensary for their prompt resignation, as a mark of their reprobation of this disgraceful regulation.

Since the above was written, we find that those candidates who put forth their offers in the newspapers, have thought proper to recal them. This is the only prudent step they could take, though it will not entirely retrieve their character, since it was the counsel of their friends, or the indignant voice of the profession, that caused the recantation. Nevertheless, they have now acted right—and perhaps their first step, imprudent as it was, will be beneficial to the right cause, by more completely deterring all respectable men from following in the same line. A period has arrived, when medical men should come forward firmly to vindicate their rights, and free themselves from the despotic insolence of non-professional governors, and committees of charitable institutions. It is most provoking to see these petty tyrants domineer over and annoy the medical officers, who have all the trouble, anxiety, and responsibility. The Aldersgate governors have received a lesson which they will not readily forget! We strongly advise the medical officers of other charities, especially in the country, to imitate the example of their metropolitan brethren, and insist on proper laws, or resign their places. After what has happened here, there will hardly be found any respectable members of the profession who will truckle to committees, and thus degrade the corps to which they belong.

---

### II. Royal College of Surgeons.

The Council of the College has sent forth a "*statement*," or exposition of the past and present state of the College, in reference to finances, library, museum, lectures, &c. by which it appears that the institution, since 1800, has been in a very flourishing condition. At the above date the property of the College did not exceed fifteen thousand pounds sterling, whereas the average receipts of the College for the last three years have amounted to upwards of eleven thousand pounds per annum! The average expenditure has been rather above eight thousand pounds per annum. The foregoing receipts are chiefly made up from the diplomas granted to surgeons—but as there is no law to compel the student to take out a diploma on commencing practice, the revenue from that source is somewhat uncertain.

The museum requires a considerable sum annually, both for conservation, and for additions. It is open to all properly introduced visitors from ten till four o'clock, on Mondays, Wednesdays, and Fridays—and to scientific foreigners every day. The library appears to be rapidly increasing, and already contains 16,000 volumes of standard works, ancient and modern. It is open every day from ten till four o'clock, for all members of the College and their articled students—except during the month of August.

" When the College received its Charter from the Crown, it derived no assistance of any kind from the other branches of the legislature: The Charter was simply permissive, allowing the Court of Examiners to examine those who might voluntarily present themselves, but giving them no legal authority whatever, to compel practitioners in surgery to obtain their Diploma, nor to prosecute those who took upon themselves to practice without it. The College, therefore, possessing no other influence than that of opinion, was left to rest altogether on its own character. Under these circumstances, it would never have advanced to its present state of prosperity, if it had failed to obtain the confidence of the profession and the public; and the best proof that it has succeeded in this object, is to be found in the increased and increasing number of the Members. In the first two years after the establishment of the College, the Diploma was granted to 300, and in the last two years to not fewer than 770 Members."

These facts speak very strongly in favour of the general estimation in which the College diploma is held by the public; but they by no means prove that the said College, in respect to its government, is in equal estimation with the profession. The general public know nothing, and care nothing about the management of affairs in Lincoln's-Inn Fields—all they know is, that the profession of a diploma from the only constituted surgical authority in the kingdom, is an indication that the individual has received a proper surgical education, and undergone a regular examination. When the council tells us that they possess no power to compel students to become members of the College, they do not exhibit an overplus of candour. The student *is* obliged to become a member, if he has any ambition to succeed in his practice, either in town or country. In town, he is not eligible to fill any public situation in hospitals or dispensaries, without the diploma—and, in the country, he well knows that a contemporary, with the diploma in his pocket, would soon expose him to the distrust of the place where they reside. Every body knows that it is not from any love to the College, or admiration of its government, that students present themselves for examination; it is from the necessity of having a testimonial of this kind, in practice—and there is no other source whence it can be procured, in this country, than the College of Surgeons. Let not the council of the College, then, lay this flattering unction to their souls—or rather let them not expect to convince the profession, by such a piece of sophistry, that the institution is on the very best establishment, and guided by the best and wisest principles. While the principle of self-election is the grand and ruling principle of the College, it will be looked upon as a close borough, and consequently a corrupt one. It will never give satisfaction to the great body of its members, while the old leaven of corporation monopoly pervades the mass, and poisons, or is supposed to poison, the vital principle of general utility.

We are no enemies to the College of Surgeons—on the contrary, we think it has done much good, and that it will do much more. We would only admonish the rulers of that body not to shut their eyes to the change of times and circumstances around them; but to make every practicable reform in good time.

---

### III. MEDICAL REFORM.

### *College of Physicians.*

"TO THE HONOURABLE

THE COMMONS OF THE UNITED KINGDOM OF GREAT BRITAIN AND IRELAND IN PARLIAMENT ASSEMBLED,

The Petition

OF THE

UNDERSIGNED PHYSICIANS, PRACTISING IN LONDON,

HUMBLY SHEWETH,

THAT the Charter of the Royal College of Physicians of London was granted by Henry the Eighth, for the advancement of Medical Science, and for the protection of the Public 'against the temerity of wicked men, and the practice of the ignorant.'

That six Physicians were named in the Charter, who, together with all men of the same Faculty, then resident in London, were constituted One Body, Commonalty, or perpetual College.

That the perpetuity of the College was to be kept up by the future admission of all men of the same Faculty into the College.

That several of the six Physicians named in the Charter, studied at, and possessed Degrees from, foreign Universities; and that no distinction is mentioned, as regards the University where a Physician may have obtained his degree.

That all Physicians entitled to Practice in London, are equally entitled, under the Charter, to Admission to the Fellowship of the College.

Your Petitioners are prepared to show, that Bye-Laws have been framed, and long acted upon, by the College, which are directly opposed to, and in violation of, the letter and meaning of the said Charter.

That the Physicians practising in London, are invidiously divided, by the Bye-Laws of the College, into Two Orders: one is denominated Fellows; the other, constituting by far the majority, is designated (and by implication degraded) by the term Licentiates.

That the Fellows have usurped all the corporate power, offices, privileges, and emoluments, attached to the College; that the Licentiates do not participate in these benefits, but are illegally excluded from all the offices, and any share in the management of the Corporation; and so far is this principle of exclusion carried, that the Licentiates are not even admitted to the Library or Museum of the College.

That there exists no foundation in the Charter, or in the Acts confirming it, for such distinction of orders, and consequent exclusion from all privileges.

That, according to one of the Bye Laws, no Physician can claim admission as a Fellow, unless he has graduated, or been admitted *ad eundum* at the Universities of Oxford or Cambridge, where medicine is imperfectly taught; while Physicians who have graduated at other British or Foreign

Universities, celebrated as Schools of Medicine, are unjustly excluded from the Fellowship, by this obnoxious Bye-Law.

That the College was admonished from the Bench, by the Lord Chief Justice Mansfield, to amend their Bye-Laws, in reference to the admission of Licentiates into the Fellowship: that, influenced by this censure, the College framed other Bye-laws, deceptive in their character, which, whenever they have been acted upon, have tended still further to depress and injure the order of Licentiates.

That the College demand and receive a large sum of money from the Fellows and Licentiates, for the supposed privilege of practising as Physicians, within a circuit of seven miles round London, and that they do not and cannot protect them in this privilege.

That the Graduates of Oxford and Cambridge are obliged to be Members of the established Church of England, and, consequently, all Dissenters are excluded from claiming the Fellowship: this, your Petitioners consider as a grievous injustice, and an act of intolerance unbecoming the present age.

That these invidious Bye-laws, made in the spirit of Corporate Monopoly, have involved the College in continued litigation, and created a jealousy between the Fellows and Licentiates discreditable to the Members of a liberal profession.

That your Petitioners, with deference, submit, that the College of Physicians, as at present constituted, is wholly inadequate to the due regulation of the Medical Profession in this Country and the protection of the public;—and further, that the Charter of the College in no way provides for the practice of Physicians in the several Counties of England and Wales.

Confiding in the wisdom of Parliament, your Petitioners therefore

Pray, that your Honourable House will institute such inquiry into the state of the Medical Profession in this Country, and the College of Physicians in particular, as will lead to the framing of laws, by which the evils complained of may be removed.

And your Petitioners will ever pray, &c.

| | |
|---|---|
| Gilbert Blane | Whitlock Nicholl |
| Henry Clutterbuck | A. T. Thomson |
| George Birkbeck | John Sims |
| W. Somerville | James Copland |
| Alexander Morison | George Gregory |
| Thomas Brown | J. C. Somerville |
| Alex. Henderson | James Bartlet |
| Charles F. Forbes | John Webster |
| Charles Locock | Tho. Harrison Burder |
| Neil Arnott | Thomas Davies |
| Roderick Macleod | T. Southwood Smith |
| John Vetch | David Barry |
| W. Gairdner | Charles Holland |
| William Russell | John Foley |
| Hugh Ley | Francis Boot |
| James Clark | R. M. Kerrison |
| Robert Lee | C. J. Roberts |
| Marshall Hall | William Stroud |
| William Whymper | James Johnson |
| Thomas Hodgkin | Edward Rigby |
| C. J. B. Williams | Robert Richardson |
| Alexander Tweedie | G. G. Sigmond |
| Henry Davies | James Hope |
| J. W. Crane | A. T. Holroyd. |
| Theodore Gordon | |

It is needless to tell our readers how long and how often we have conjured the College of Physicians to revise their laws, and put themselves in some degree of accordance with the spirit of the age, before a burst of indignation came from their helot licentiates in London, and their brethren in general throughout the kingdom. But, learned as the collegiates are, they did not understand, or they would not believe, the well-known truth—

"Quem Deus vult perdere prius dementat."

No! Like the Jews in the Temple, nothing, could persuade these ELECT that their sacred, time-hallowed, king-blessed, monk-erected edifice was vulnerable in any quarter, fallible in any point, or capable of improvement, even in the most trivial particular! All was Millennium with the College—they were the heaven-born Esculapii of England —who sucked in medical science, and practical experience, with their Greek and Latin on the Cam and the Isis! The reign of Tory corruption and Corporation monopoly was never to have an end—the statutes of Henry the VIII. were as pure, as indissoluble, as those delivered on Mount Sinai, amid thunders and lightnings, to the wondering Israelites. It was incredible that a band of fifty Helots should stand forth in London alone, and dare to petition the legislature for a dissolution of their chains! Still, even this significant symptom will have little or no effect on the College. The bigotted portion of the collegiates will listen to no reason. You might as well preach liberty and equality to the Autocrat of the bears, as propose a single liberal measure to the high-church party in the College. They consider themselves as the nobility of the profession, and to take away from them the insignia of their order, would be to deprive them of the only distinction between themselves and their humbler brethren. But we are wasting words on a part, when the whole stands in need of reconstruction. It is of comparatively trifling consequence, what becomes of the College of Physicians. If they do not open their doors to the Licentiates, and to all qualified physicians, these physicians will not trouble themselves about entering the portals of the College at all. In the present state of things, and, *à fortiori,* in future, the College possesses and will possess no legal power over physicians settling in the metropolis ; and they are really fools who shall give themselves any trouble about taking out an insult (which is the proper name of the *license*) at the expense of 57 pounds sterling. The College never again will dare to prosecute a regular physician (and the irregular one is their protegé) for the practising in London without a license—that is certain. The only anchor of hope which they have left, is the refusal to consult: but this anchor will soon drag the bottom, and refuse a stay to the drifting ship. The public will shortly become acquainted with the reasons on which these refusals are based, and will disregard them accordingly. Neither have the candidates for metropolitan practice much to fear from the ban of the College as to consultations. Little need they expect from their patronage at any time, and little need they fear their refusals to participate in the Guinea trade, when the fee presents itself. With such a list of Licentiates as the metropolis now presents, the non-licensed physician (always provided that he is duly qualified) can experience no difficulty. He has only to produce the College list itself, and tell the patient that the *minority* refuse to meet him on medico-political grounds; while the overwhelming *majority* are ready to consult with him—and the charm of the BAN is broken at once.

But again and again we exhort our brethren of every denomination, from the Pentland Frith to the Isle of Wight—from the Shannon to the Humber, to forego all party disputes, to waive all corporate interests, and join in the "universal prayer" for PARLIAMENTARY INQUIRY. This is the measure from which we have every thing to hope—this is the test from which the enemies of reform have every thing to fear! Let the physicians, surgeons, and general practitioners of every city, town, and village of the united kingdom, forward petitions to the next parliamentary assembly, even if they contain but three words "*Medical Parliamentary Enquiry*"—and the object will be obtained. Fortunately for our cause, we only want the light to be let in on darkness, and the deformity of our laws respecting medicine, will be apparent to the meanest capacity. Our Irish brethren are bestirring themselves, and petitions are getting ready in various parts of the sister Isle. It is time for the English faculty to shew activity.

We think it would be premature, if not injurious, to be perplexing the minds of the profession and society at large, by proposing panaceas and nostrums for the disease, before the history and symptoms are fully delineated before the legislature. If a parliamentary inquiry be granted, the Committee or Commissioners will have ample means of drawing from individuals, not only the nature of the evil, but the probable remedy for it. Then will be the time for specific proposals. Meantime, during the recess, every member of the profession, who is not sunk in apathy, and perfectly contented with things as they are, should endeavour to explain to those members of Parliament, with whom he may come in contact, the chaotic state of our medical constitution, and the urgent necessity there is for a thorough—indeed *radical* reform. This is a precaution which should not be neglected. More might be done in this way than by interminable lucubrations in our medical journals, which never reach the public ear.

Mean time an association is organized in the metropolis, which will prove the nucleus, or the punctum saliens, for similar associations in the provincial cities and large towns. Nearly a hundred of the most talented, active, and liberal physicians of London, have entered into league, with the certain prospect of being joined by nine-tenths of the physicians of the United Kingdom, to further the cause of medical reform, by every possible means. Their proceedings will be made known to the public—and we have little doubt that their voice will be heard in the next Session of Parliament.

As might be expected, some of the crawling sycophants, who would go on their knees from Piccadilly to Pall Mall East, kissing the mud and making an abject salam to the President at every step, for admission into the temple, are hanging back! ! Yes! These are the men who would sacrifice the whole profession for the sake of having "FELLOW," attached to their names !! We trust that the day is nearly at an end, when the Machiavelian practice of crushing the spirit of the Licentiates, by plucking from them one or two annually, to swell the tail of the FELLOWS, shall have operative influence. Those who *hereafter* accept such degraded honours, should have the finger of scorn pointed at them, from one end of the kingdom to the other. They will be traitors to the cause of medical reform, ten thousand times more abject than the renegade Christian who tramples on the Cross, denies his Saviour, and embraces Islamism, for a few thousand piastres!

If we had a hundred tongues, we would reiterate the word "PETITION." There can be no difficulty in such a procedure—no excuse for not joining in the good cause. The Licentiates of London have led the way — not for themselves alone, but for the profession in general. Their example should be followed from one end of the kingdom to the other—not by any one class in particular; but by physicians, surgeons, and general practitioners united. The burthen of the prayer should be, that medical polity in this country, is a chaos of clashing interests and injurious regulations, which nothing but a Parliamentary inquiry can reduce into order, for the benefit of the community. It is needless to expect any salutary measures from either of the three corporate bodies now existing. They are all and each working for their own separate interests, and care not a farthing for the good of the profession at large. The work of reform must be taken out of their hands entirely, and placed in those of our legislators. If petitions are not presented at the very earliest period of the next Session, from the whole profession, we shall have a ROYAL COMMISSION—a packed Jury, constructed under the influence of a court faction—and then adieu to all wise legislation!

